# Facilitating Integration Through Team-Based Primary Healthcare: A Cross-Case Policy Analysis of Four Canadian Provinces

**DOI:** 10.5334/ijic.5680

**Published:** 2021-11-08

**Authors:** Alexandra Lukey, Sharon Johnston, Stephanie Montesanti, Catherine Donnelly, Paul Wankah, Mylaine Breton, Isabelle Gaboury, Simone Parniak, Caille Pritchard, Shannon Berg, Karin Maiwald, Sara Mallinson, Lee A. Green, Nelly D. Oelke

**Affiliations:** 1 School of Nursing, University of British Columbia, Okanagan, CA; 2 Department of Family Medicine, University of Ottawa, Institut du Savoir Montfort, CA; 3 School of Public Health, University of Alberta, CA; 4 School of Rehabilitation Therapy, Queen’s University, CA; 5 Department of Community Health, Université de Sherbrooke, CA; 6 Department of Family Medicine and Emergency Medicine, Université de Sherbrooke, CA; 7 Occupational Therapy, Faculty of Rehabilitation Medicine, University of Alberta; Associate, Centre for Clinical Epidemiology and Evaluation (C2E2), Vancouver Coastal Health Research Institute; 8 Occupational Science and Occupational Therapy, University of British Columbia, CA; 9 Research Department, Interior Health; Adjunct Professor, Faculty of Medicine, University of British Columbia, CA; 10 Health Systems Evaluation and Evidence, Alberta Health Services; 11 Department of Community Health Sciences, Cummings School of Medicine, University of Calgary, CA; 12 Department of Family Medicine, University of Alberta, CA; 13 Rural Coordination Centre of British Columbia, CA

**Keywords:** Integration, Integrated health services, Interprofessional teams, Primary healthcare, Primary care, Team-based care

## Abstract

**Introduction:**

Team-based care can improve integrated health services by increasing comprehensiveness and continuity of care in primary healthcare (PHC) settings. Collaborative models involving providers from different professions can help to achieve coordinated, high-quality person-centred care. In Canada, there has been variation in both the timing/pace of adoption and approach to interprofessional PHC (IPHC) policy. Provinces are at different stages in the development, implementation, and evaluation of team-based PHC models. This paper describes how different policies, contexts, and innovations across four Canadian provinces (British Columbia, Alberta, Ontario, Quebec) facilitate or limit integrated health services through IPHC teams.

**Methods:**

Systematic searches identified 100 policy documents across the four provinces. Analysis was informed by Walt and Gilson’s Policy Triangle (2008) and Suter et al.’s (2009) health system integration principles. Provincial policy case studies were constructed and used to complete a cross-case comparison.

**Results:**

Each province implemented variations of an IPHC based model. Five key components were found that influenced IPHC and integrated health services: patient-centred care; team structures; information systems; financial management; and performance measurement.

**Conclusion:**

Heterogeneity of the implementation of PHC teams across Canadian provinces provides an opportunity to learn and improve interprofessional care and integrated health services across jurisdictions.

## Introduction

To achieve significant improvements in health system performance, strengthening primary healthcare (PHC) is important [1]. PHC, defined as “meeting people’s health needs through comprehensive promotive, protective, preventive, curative, rehabilitative, and palliative care throughout the life course [2, para.6], is the cornerstone of a high performing integrated health system [1,3] Integrated health services include “the management and delivery of health services so that clients receive a continuum of preventive and curative services” based on needs, over time, across health system levels [4, p. 1]. Integrated health services also encompass cross-sector services such as social services, housing, and education addressing the social determinants of health [2]. Integrated health services in PHC can be achieved through interprofessional primary healthcare (IPHC) teams composed of health and social care providers [5].

Over the last two decades, Canadian PHC has undergone a significant shift towards integrated interprofessional team-based care that is patient-centred [6]. IPHC teams include two or more healthcare providers who formally work together to care for a patient [7]. The main characteristics of an IPHC model includes care that is coordinated and integrated within the team and across the health system as a whole [8,9]. Team members may be co-located in an interprofessional clinic setting such as Family Health Teams (FHTs) in Ontario or not and supported by overarching policies and clinic networks which promote team-based care such as Primary Care Networks (PCNs) in Alberta [9,10].

The establishment of IPHC was an objective of Canadian national reform in the early 2000’s [7,11,12]. IPHC was initiated by a First Ministers’ agreement which led to government investment in the Primary Health Care Transition Fund of $800 million where team-based care was to become the cornerstone of IPHC in Canada [10,13,14]. The fund supported provinces to introduce new approaches to delivery over a six-year period (2000–2006). The goals of transitioning to an IPHC model were motivated by health system pressures, including increased focus on chronic disease management, preventative care, and physician shortages [15,16]. Although initiatives were Canada-wide, responsibility for implementation and delivery was left to provinces [17], creating wide variation in the interpretation of policies that led to varied priorities and investments to achieve the goal of IPHC [18].

With ongoing PHC transformation in Canada, further evidence is needed to inform improvements in integrated IPHC. The purpose of this paper is to describe how the content of policy documents are similar or different across four Canadian provinces, British Columbia [BC], Alberta [AB], Ontario [ON], and Quebec [QC], with regard to IPHC teams and integrated health services. Analyzing content, facilitated a better understanding of how IPHC teams have been implemented in the different provinces to support integration through policy documents. A theory driven search and review of policy documents was completed to identify macro (provincial) and meso (e.g., health authority) policy documents relevant to IPHC within the context of Canadian PHC reform over the last decade.

## Methods

Key PHC policy documents were identified in target provinces through systematic online searches and key stakeholder knowledge. We used Walt and Gilson’s *Policy Triangle* [19] to explore the interrelationship and interaction among four main components of policy-making which include actors (individuals, groups, and organizations involved, and their interactions with one another), processes (how policies are formulated and implemented), context (socio-political, cultural, economic, and health system setting), and content (the policy’s substance and details such as objectives, service models, operational guidelines, and implementation plans) within the policy documents from different provinces. Health policies are developed in the complex interactions between the content of policy, the actors involved, context and processes. The policy triangle framework was used to organize and think systematically about how these four components might affect policy decisions on IPHC and integrated health services. We used the *Ten Key Principles for Successful Health Systems Integration* [5] to identify and extract policy content related to integration. By using a framework driven content review and data extraction across participating provinces, we were able to explore similarities and differences in how IPHC teams and integration principles were represented in policy documents. It is important to note that the extent to which policy documents have been implemented and/or evaluated in practice are beyond the scope of this study.

### Inclusion of policies for analysis

In each province, policy documents were identified through knowledge users on our team (provincial policy and decision-makers) and via Google and Google Scholar searches. Search terms included: “primary health care” OR “primary care” AND “[province name] AND “team” OR “integrat*” OR “complex patient*” OR “patient engagement.” See Appendix A for detailed inclusion and exclusion criteria. While both provincial and regional policy documents were sought, most documents identified were provincial policies. Documents were collected for analysis throughout 2018/19, although ON undertook a search in 2020 given the new model of care (Ontario Health Teams) that was being initiated. Examples of policy documents included were strategic direction documents, business plans, PHC framework documents, and advocacy documents. These documents included a mix of formal policy documents that had been enacted and sponsored by provincial governments and regional organizations and guidelines and frameworks that provided support and advocacy for PHC redesign.

### Data extraction and analysis

We analyzed 100 policy documents from four provinces (BC n = 12, AB n = 18, ON n = 55 [15 provincial and 40 regional documents], QC n = 15) (see Appendix B for full list). The team developed focused extraction templates to guide the review of included policy documents and extract data for analysis. Data from policy documents were extracted into a single matrix (Appendix C) based on the study frameworks [5,19]. Data were then coded and examined for coherence and duplication across policies within and between categories in the matrix. The policy triangle was also used as framework for extraction and analysis of identified policy documents. All obtained provincial documents were read, and data extracted focusing on the content, the context, the process of policy development as well as the actors involved in developing the policy related to IPHC teams and integrated health services. Using the matrix of data extracted from policy documents each province created a descriptive case-study synthesis that summarized key findings. These were discussed, refined, and agreed upon by research team members for each province, including knowledge-users. These provincial case-studies were then used for comparative analysis. Comparative analysis was undertaken by a research assistant (AL) supported by the principal investigator (NDO) and reviewed by team members from each province for accuracy of the content as well as the interpretation of policy documents.

## Results

The cross-case comparison of the four Canadian provinces yielded common and divergent themes on the content of policy documents on IPHC teams and integrated health services. ***Table 1*** shows our results from the *Policy Triangle* analysis [19].

**Table 1:** Policy actors, context, and process by case.


PROVINCE	PRIMARY ACTORS	CONTEXT	PROCESS

**BC**	BC Ministry of Health Doctors of BC General Practice Service Committee	Five Regional Health Authorities (RHAs) Ministry of Health oversees management of health services Primary care teams began in 2008 as Integrated Health Networks, through RHAs and BC Medical Association Primary Care Networks (PCNs) implemented (2018)	Policy documents development processes not standardized Goal to transform family physician practices and primary care clinics into team-based Patient Medical Homes, linked and connected with a team-based PCN and RHA A Medical Health Officer designated for each PCN for regional/provincial connection

**AB**	Government of Alberta (Alberta Health [AH]) Alberta Health Services (AHS) Alberta Medical Association (AMA)	Trilateral Master Agreement signed by AMA, AH and Regional Health Authorities (2003) PCN model adopted (2005) with 80% physicians attached to PCNs (2016); most experience with PCN model Single province-wide health authority implemented (AHS) (2009)	Policy documents developed by AH; implemented by PCNs as a condition of grant agreements PCNs are joint ventures between family physicians and AHS, accountable to AH PCNs funded by AH Physicians’ practices largely use fee-for-service model

**ON**	Ministry of Health (MOH) Health Quality Ontario (HQO) Ontario Primary Care Council (OPCC) Local Health Integrated Networks (LHINs) Association of Family Health Teams of Ontario (AFHTO)	Interprofessional teams in Community Health Centres for 40 years Various primary care models introduced (2000–2010) Interprofessional Family Health Teams (FHTs) introduced (2006) Innovations in remuneration models such as Enhanced Fee-For-Service models, capitation models, salary models, and various incentives and bonuses Change in government with reform to dismantle LHINs and introduce Ontario Health Teams (OHTs) (2019)	MOH implements and evaluates policy guidelines for the province LHINs identified as catalyst for improving integration at the local level through Integrated Health Service Plans for regional governance based on provincial guidelines MOH established HQO to evaluate Ontario health system, including primary care; external evaluations commissioned of primary care models, particularly FHTs Policy is shifting from identifying primary care as a key enabler of integration

**QC**	Ministry of Health and Social Services (MHSS) of Quebec Health and Social Services Centres (HSSC) Integrated Health and Social Service Centres (IHSSC) College of Physicians of Quebec Quebec Nurses Association	Introduction of interprofessional (physicians and nurses) Family Medicine Groups (2001) Creation of 95 HSSC through administrative mergers of hospitals, community service centres and long-term care facilities (2004) Creation of 22 IHSSCs through administrative mergers of HSSCs, rehabilitation centres and youth centres (2015) Social workers introduced into Family Medicine Groups (2016)	High level policy documents developed by government and implemented by MHSS Regional health and social service agencies, HSSCs and IHSSCs adapt policies to local context/priorities. Regional health and social service agencies, HSSCs/IHSSCs accountable to MHSS through regular reports and data submission Leveraging administrative mergers (HSSCs/IHSSCs) of public healthcare organisations to enhance inter-organizational connectivity through health networks.


Based on the *Ten Key Principles for Successful Health Systems Integration* [5], we outlined key components of integrated health services through IPHC identified from the data from policy documents. We focused on five of the 10 key principles [5]. *Patient focus standardized care delivery through interprofessional teams, information systems, financial management, and performance management* were chosen as principles for this analysis for their frequency and depth of discussion in the policy documents across provinces. While principles of *comprehensive services across the care continuum, geographic coverage and rostering, organizational culture and leadership, physician integration, and governance structure*, were present in many policy documents, they were less prominent themes. Our analysis was focused on the most prominent themes of integration for a more comprehensive comparison of the impact policy document content had on the development of IPHC teams in a Canadian context.

## Components of Integrated Health Services through IPHC teams

### Patient Centred Care and Engaging Patients in Policy Document Development

The theme of patient-centred care was identified in provincial documents as an integral component to integrated health services for the purpose of improved coordination of services, accessibility, and patient experience. Patient-centred care was included in the Patient Medical Home model [20,21] used by some provinces (e.g., BC, QC). One element of patient-centred care entailed providing care as close to home as possible [22,23,24]. Policies also supported providing home-based IPHC services to patients; particularly patients with complex care needs. In both AB and BC, PCNs’ interprofessional teams worked together to provide care specific to community/population needs. PCNs in BC were governed at a local level, which allowed more flexibility of local leadership to meet the needs of the specific population they served [25,26].

The Local Health Networks of Quebec tailored services to their population by stating that “characteristics of the population and the communities (cultural, linguistic, etc.) that compose it are taken into account in the response to needs” [27, p. 21]. Similarly, the 2012 Ontario Action Plan for Health Care described itself as “obsessively patient-centred” [27, p. 7] and a subsequent primary care measurement framework released by Health Quality Ontario in 2014 listed measurements for patient centeredness, including respect for patient and family values, cultures, needs and goals [27, p. 37]. To align with patient-centred care, a province-wide process for tracking and addressing patient concerns was recommended in AB to contribute to continuity of care and reduce duplication of concerns and errors [28].

Despite creating patient-centred policies and practices, there was significant variation in how patients were involved in the development of policies. Examples of patient involvement in policy document development included a patient representative on PCN Boards in AB [29] and the ON Ministry of Health seeking public feedback on a policy proposal [30]. Due to the variation in number and level of policy documents in each province, we were unable to draw conclusions across provinces; however, patient engagement in policy development was a topic mentioned in a number of policy documents.

### Team Structures

Team structures to support integrated health services varied across provinces, given the different models of IPHC teams. In AB, PCNs were the main model used to promote and incentivize team-based IPHC. In 2003, PCNs were created through an agreement between the Alberta Medical Association (AMA), the Ministry of Health, and the regional health authorities (which were eventually merged into Alberta Health Services). PCNs were supported by funding for infrastructure, quality improvement, governance, and team-based care [29]. In ON, Family Health Teams and Community Health Centres as well as several other less common models were predominant models serving over one quarter of the population over the past 15 years. In the past two years, however the newest health system reform introducing ON Health Teams sought to build integrated health systems encompassing all sectors of care, including PHC teams, serving specific patient populations [31]. In QC, Family Medicine Groups (FMGs) were the primary structure for care delivered by IPHC teams [32,33]. Since 2002, more than 330 FMGs have been implemented across QC. BC recently initiated their PCN model with geographically linked services including family practices, community governed health centres and health authorities [34]. These models, albeit different in each province, all create infrastructure for IPHC teams to operate in the existing PHC system to improve integrated health services.

While some provinces (BC, ON and QC) incorporated physical co-location of their IPHC teams, provinces also included geographic networks of clinics and providers for team-based care such as the PCN model in BC and AB [35,36]. Co-location required physical space for teams to work together and collaborate [36,37]. For example, Urgent Family Care Centres in BC were described as a hub for patients to receive care from a variety of professionals [34].

One policy document recommended the ratio of non-physician team members to a physician for maximum team functionality in the Alberta PCN model was 3–4:1 [38]. Ratios, where reported in this analysis, differed significantly from this recommendation. AB reported a 1:1 ratio [22], however, the accuracy of reporting was impacted by the limitation of the number and level of policy documents focusing on ratios. In QC, the number and type of other providers was based on the number of enrolled patients within the FMG [32]. Frequently mentioned in policy documents was that many non-physician providers have historically not optimized their scope of practice in IPHC [28,39]. The lack of clarity in team member roles and in leadership responsibilities contributed to the lack of team members working to their optimal scope of practice [38,40]. The support for interprofessional team members to optimize their scope of practice including role clarification and team structure, was stated to be a key component for effective IPHC in policy documents [39,41,42,43,44]. Finally, collaboration and co-creation of structures and processes with professional associations were discussed in policy documents as a critical step to creating buy-in and support for new directions such as the transition to improving integration through IPHC teams [28,45].

### Information Systems

There was significant variability in the use of information technology, ranging from a province-wide integrated system linked with lab results and prescriptions (Dossier santé Québec) to significant province-wide information sharing incompatibility (ON, BC and AB). Where interoperability challenges were discussed, a single comprehensive electronic health record (EHR) for each patient was stated as a goal to overcome the current fragmentation of health information [23,38]. For instance, due to EHR incompatibility, while regions may be able to share information, rural or other geographic locations may be excluded [46]. In AB, several information system initiatives have been launched to streamline communications between providers to improve outcomes for patients, especially during care transitions such as NetCare and Connect Care [47].

### Financial Management

No province had a single compensation model for PHC and most often there was a separation between physician compensation and compensation for other team members. The most common remuneration model for physicians discussed in policy documents was fee-for-service compensation. In one policy document, it was highlighted that fee-for-service did not incentivise physician participation in an interprofessional care team [38]. Furthermore, fee-for-service negatively impacted the care of patients with complex needs where multiple issues per visit were not addressed [38]. In ON, FHTs used a blended model of capitation and fee-for service [48]. In QC, the predominant model was FMG with publicly funded private clinics. Fee-for-service with capitation based on the enrolled number of patients was used to support IPHC teams in QC [49,50].

The concept of value-based compensation was introduced in BC, an example of shifting towards compensation based on quality indicators and panel size rather than fee-for-service funding [34]. Overall, the policy documents included in this study, showed development towards innovative funding models of team-based care; however clear guidelines or recommendations for funding IPHC teams was limited. Financial support and incentivization were found throughout the policy documents with variation in levels of discussion. Policy documents did suggest that conversations on compensation and incentivization of IPHC teams were essential and needed in future work.

### Performance Measurement

Performance management was frequently mentioned as a crucial part of improving integration of health services through IPHC teams [28,51,52]. In BC, the need for quality improvement was addressed in PCN documents [34] but specific outcomes and indicators were not yet published [35]. In QC, a provincial law (Bill 20) imposed a province-wide target of 85% attachment with the threat of substantial remuneration cuts to providers if the target was not met. Performance measures used to date were based more on accountability of resources invested in FMG. In AB, quality improvement goals were described to advance team-based PHC. Several AB policy documents outlined possible evaluation methods and standards for measuring performance [53,54]. In 2014, Health Quality Ontario developed a measurement framework for primary care [55]. The Ontario Primary Care Council, comprised of the Associations of FHTs of Ontario and Ontario Health Centres, among others, developed a comprehensive set of indicators specifically for primary care [56]. Many of the policy documents mentioned the Quadruple Aim [57] and attempted to align conversation about performance measurement to the same. Overall, performance measurement varied in context and level across provinces.

## Discussion and Recommendations

The value and importance placed on integrated health services delivery in PHC is evident in these four Canadian provinces. How the integration of health services is supported through IPHC teams and put into practice is less clear from the policy documents reviewed. What we do know is that IPHC teams have been introduced in all provinces, with variability in their implementation. In some provinces, such as ON and QC, IPHC teams were implemented earlier, with AB starting a few years later, and BC only recently implementing IPHC teams through PCNs.

There is a need for integration of IPHC teams at both the individual team and at the health system level. While individual integration of IPHC teams may improve overall health services integration, system level change is also necessary to embed IPHC teams in the system overall to facilitate integrated health services delivery for patients. Policy at a provincial level may facilitate the necessary environment for IPHC teams to function through governance direction and funding incentives. Content in the policy documents did not explicitly address this issue of the necessity of integration at both levels. Yet, discussion is present in policy documents that address challenges both within an individual team and at the macro system level.

Our analysis looked for the presence or acknowledgement of various actors, specifically patients including family members and caregivers, in the policy documents. All provinces provide evidence of the importance of patient engagement in their discussion of policy document development processes. However, many policies lack any mention of patient involvement or mention involvement but provide no details as to how they were involved. Further, what was less clear is how they are engaged over the longer term in policies for integration through IPHC teams at a provincial level. Suter et al. [14] highlight this gap between patient-centred policy and patient representation in the process of developing policies. A better understanding is required, at regional/provincial levels, of how patients have been engaged in policy development, evaluation on the impacts of engagement, social interactions and problem solving in practice, and understanding if engagement has been meaningful to both patients and policymakers. To inform the policy-making process, engagement of diverse patients is needed for a better understanding of the different experiences patients have and their perspectives for better healthcare and health. More work is needed to examine patient engagement in the development of provincial/regional policy to improve IPHC and the delivery of integrated health services [58].

Team-based care with clearly defined roles can be a key component to effectively functioning teams to support integrated health services [5,15,59]. Both systemic barriers, as well as resistance of professional organizations, impacts the ability to address roles and responsibilities on teams [14]. Team composition is most often not discussed in policies and rightfully so, as the interprofessional mix should be determined by the needs of the population served as noted in a number of policy documents across provinces. There is also limited discussion about how population needs may be assessed and how this would determine IPHC team composition to ensure needs are being met for better integrated care.

Finally, team structure in some policies discuss co-location of IPHC teams. There is wide-ranging support for having a physical co-located space for teams, although this may not be feasible or realistic given Canada’s dispersed geography. That said, the recent move in the use of virtual care during the COVID-19 pandemic suggests collaboration rather than co-location may be the relevant next focus. Co-location is frequently discussed in the literature as important to the operational development of team-based care including enabling team-communication [9,14,60]. Although co-location may be seen as important, provincial policies show a mix of co-located IPHC teams and those that are not. What is not well understood is how this impacts the quality of integrated health services, and what tools (e.g., compatible health records, virtual means of communication) are needed to enable better integration through IPHC teams that are not co-located. Future research is needed on the impacts of virtual team-based care versus co-location.

An important theme highlighted in the policy documents that supports integrated health services is access to interoperable EHRs accessible to all IPHC team members. This is especially crucial for teams that are not co-located and more so in the current context of virtual care being delivered by virtual teams, to facilitate continuity of care and seamless provider communication [61]. In fact, patients may view this as a key measure of the health system’s performance [62]. In this review, QC was the province to focus most on creating a provincially compatible EHR for lab results, although strides have been made more recently in AB to innovate health information systems. Information systems are mentioned frequently in IPHC policy documents, yet many recommendations, as noted by Steele Gray et al. [61], made in policies have not yet been realized. Continued work in this area is needed for both improved integration of team-based services and streamlined communication between providers and providers and patients.

With regards to the context under which these provincial policy documents are developed, several drivers were found to influence integrated health services through IPHC teams across the four provinces. One key obstacle in the development of integrated health services including IPHC teams continues to be identified as financial remuneration models that do not always incentivize team collaboration as supported by both policy document analysis and the literature [8,10]. Remuneration is addressed at varying levels across the policy documents analysed with different compensation models and combinations of models being used across the four provinces. The complexity of compensation is highlighted with variations in financial management, including payer, payment model and incentives. There are extensive recommendations, however, specific direction for team compensation is still unclear across provinces. Furthermore, there appears to be a lack of evidence used in articulating remuneration models for IPHC in the policies reviewed. Wranik et al. [60] found PHC compensation models internationally tend not to be based on evidence. The qualities of compensation that inhibit high functioning interprofessional teams include funding that is linked to physician activities only, lack of initial investment to support the clinical transition to IPHC teams, and reliance on long term financial incentivization [10,63]. Funding for IPHC teams needs to be sustainable [10,11]; too much may not be sustainable for the system or may lead to skewed incentivization, whereas too little funding may result in insufficient resources for initial team set-up and infrastructure [9,10].

Some promising evidence shows linking team compensation to team activities may support the efficacy of teams [63]. There is also low-quality evidence that shows a blended fee-for-service and capitation model may be a viable option for compensation of IPHC teams [64], such as used in ON and QC. Overall, the mixed evidence and complexity of team compensation demonstrates the need for further research to enable IPHC funding decisions to be evidence-based [65].

Team compensation also highlights issues in governance of IPHC teams. We see a combination of both private and public governance in the PHC system. This is particularly evident where private physician practice funded through fee-for-service government funding is largely the norm in most Canadian provinces [66]. Public and private governance is loosely connected in PHC systems impacting the implementation of innovative IPHC teams and integrated services delivery.

In this cross-case analysis, team-based PHC, may have been developed through the creation of new models of IPHC or by overarching policies to change practice as supported by the literature [10]. Our analysis found that provinces (AB and ON) focussed on the development of structural PHC reform had more developed evaluation processes. These provinces also have an extended history of innovation with more mature evaluation policies similar to results found by Wranik et al. [60] in other countries. However, even within both provinces, there is significant inconsistency in the quality and processes of quality reporting in the policy documents included in this analysis. Such inconsistency makes performance measurement difficult within and creates barriers to comparison between geographic regions and models of IPHC without the use of consistent Pan Canadian indicators to measure quality of PHC. Although there have been nationwide efforts to establish common frameworks of performance measurement in PHC, the use of common indicators has yet to be realized [67]. Furthermore, these indicators should be aligned with the Quadruple Aim [56].

One framework that could be helpful in addressing the challenges of measuring team performance and creating innovative change in IPHC is the learning health system (LHS) framework. While LHSs include traditional quality improvement, the learning cycles involve adaptability and cooperative and participatory leadership [68], while recognizing the complexities in health systems [69], including IPHC teams. Rather than adopting common quality indicators, the LHS model could provide a common approach to team performance measurement and innovative change in the context of the variability in population needs and shared governance of the Canadian healthcare system. Overall, there is significant work yet to be done to understand the best practices for performance to improve integrated health services through IPHC teams.

The content of policy documents included in this cross-provincial analysis expose common themes for team-based care integration. The content of the policy documents describes broad system-level change necessary for fully integrated team-based care such as patient-centred team-planning and interdisciplinary PHC incentivization. We also found pragmatic recommendations such as the need for continuity in health information sharing and shared principles for performance measurement. The Canadian shift towards IPHC teams that support integration must be supported by policy at both regional and provincial levels. A provincial-level strategy to support consistency in implementation at the regional level is necessary. However, more granular team operations such as team composition should be informed by local population needs and local leadership. Further research is needed to better understand and measure integrated health services through IPHC teams. Furthermore, we need to assess how structures and processes can support an integrated health system as a whole which then teams can effectively function within.

## Strengths and Limitations

This study includes analysis of policies over the last decade from the four largest provinces across Canada; two from the west and two from central/eastern Canada. The research has been completed collaboratively with representation from all provinces including policy and decision makers, providers, and patients.

There are several limitations to this cross-province policy document analysis. The analysis covered a broad scope of policy documents in multiple Canadian provinces over the last decade. IPHC and integration are complex topics, and all the nuances of motivations, contextual elements, and political influences are not able to be captured. Policy documents represented a snapshot in time but not exhaustive of all provincial, regional, and organizational policy documents on IPHC teams, particularly those not readily or publicly available. Availability of documents differed across the four provinces which may have contributed to differences in the policy discussion depth. Therefore, our findings must be interpreted with the limitation that we are unable to comment on the outcomes or accuracy of content of the policy documents. Interviews with policymakers may have enhanced the results of our policy document analysis. Despite these limitations, this policy document analysis will be relevant to healthcare jurisdictions at varying stages of adoption of IPHC teams and integration in PHC.

## Lessons Learned

- Implementation of IPHC teams requires integration processes at both the individual team level in PHC setting and the health system as a whole.
- More work is needed to determine optimal approaches for performance measurement to facilitate quality improvement at the clinical level and improve performance at the system level.
- Developmental LHS approaches to evaluation and QI could be considered to embed reflection and review at all levels.
- Evidenced-based remuneration models are needed to effectively support IPHC teams.
- Improved strategies for engaging patients as partners throughout the policy process (development, implementation, and evaluation) and to ensure a patient-centred approach should be supported by high quality evidence from research and evaluation.

## Conclusion

Interprofessional teams have significant potential to improve PHC integrated service delivery. This cross case-analysis found common and divergent themes across policy documents. Policies that support integrated, person-centred care while controlling costs are essential in the development of an IPHC system. Evidence-based policy supports action and decision making in IPHC for system development. The results of this study contribute to the knowledge of how these Canadian provinces have implemented IPHC teams through policy, and how policy direction has influenced similarities and differences over the last decade. This research is timely, considering the continued shifts towards IPHC both in Canada and internationally.

## Additional files

The additional files for this article can be found as follows:

10.5334/ijic.5680.s1 Appendix A. Inclusion and exclusion criteria.

10.5334/ijic.5680.s2 Appendix B. List of Policy Documents for each Provinces.

10.5334/ijic.5680.s3 Appendix C. Data Extraction Table.
