# Supplementary material for: Facilitating Integration Through Team-Based Primary Healthcare: A Cross-Case Policy Analysis of Four Canadian Provinces
Source: Int J Integr Care. 2021 Nov 8;21(4):12. doi: 10.5334/ijic.5680 (PMC8588891; doi:10.5334/ijic.5680)
Supplement: Appendix A. — Inclusion and exclusion criteria. [file ijic-21-4-5680-s1.pdf]

Appendix A. Inclusion and exclusion criteria

Inclusion	Exclusion
• Primary health care* (inclusive of preventive care)<br>• Primary care*<br>• Integration (e.g., integrated health systems, integrated service delivery, coordination of care)<br>• Patients with complex needs (two or more comorbidities, multi-morbidity and recognition of vulnerability and/or social determinants)<br>• Patient engagement (specifically related to policy & teams)<br>• Team-based care<br>• Publicly available documents<br>• 2009 - 2019	• Children/Youth population<br>• Ambulatory care, specialty care

*Both primary healthcare and primary care terms were used as they are often used interchangeably
