# Supplementary material for: Facilitating Integration Through Team-Based Primary Healthcare: A Cross-Case Policy Analysis of Four Canadian Provinces
Source: Int J Integr Care. 2021 Nov 8;21(4):12. doi: 10.5334/ijic.5680 (PMC8588891; doi:10.5334/ijic.5680)
Supplement: Appendix B. — List of Policy Documents for each Provinces. [file ijic-21-4-5680-s2.pdf]

Appendix B. List of Policy Documents for each Provinces

BC:

1. Ministry of Health: Establish Primary Care Networks2017
2. Ministry of Health: Ministry of Health: Specialized Community Services Program for Adults with Complex Medical Conditions/or Frailty 2017
3. Ministry of Heath: Integrated Health System for Primary and Community Care 2017
4. Ministry of Health: Specialized Community Services Program for Mental Health and Substance Use 2017
5. Ministry of Health: Interdisciplinary Team-Based Care 2017
6. Ministry of Health: Patient Medical Home 2017
7. Ministry of Health: Surgical Waitlist Improvement 2017
8. Ministry of Health: Primary Care Networks' Planning Guide for Preventive Care 2018
9. An Integrated B.C. Health Care System That Works for Patients Health Professionals; and Sustainability 2017
10. Setting Priorities for the B.C. Health System 2014
11. Improving Primary Health Care Through Collaboration. Briefing 1 - Current Knowledge About Interprofessional Teams in Canada 2012
12. Policy Statement. Multidisciplinary Primary Care 2011

AB:

1. Alberta Provincial Health Business Plan 2018 - 2021 (Alberta Health, 2018a)
2. Primary Care Initiative Policy Manual (Alberta Health, 2018b)
3. Primary Health Care Integration Network Transformational Road Map 2018-21 (Alberta Health Services, 2018a)
4. The 2017-2020 Health Plan & Business Plan: Year 2 (Alberta Health Services, 2018b)
5. Valuing Mental Health: Next Steps (Alberta Health, 2017)
6. Better Healthcare for Albertans: A Report by the Office of the Auditor General of Alberta (Auditor General of Alberta, 2017)
7. Alberta Health Primary Care Networks Review (Alberta Health, 2016)
8. The Patient First Strategy (Alberta Health Services, 2015)
9. Alberta's Primary Health Care Strategy (Alberta Health, 2014)
10. Continuing Care Quality Management Framework (Alberta Health Services, 2014)
11. PCN Evolution Vision and Framework (Alberta Medical Association Primary Care Alliance Board, 2013)
12. Health Business Plan 2013-16 (Alberta Health, 2013a)
13. Alberta Health Primary Health Care Evaluation Framework (Alberta Health, 2013b)
14. Strengthening Primary Health Care in Alberta through Family Care Clinics: From Concept to Reality (Alberta Health, 2012)
15. Primary Care Initiative [PCI] Evaluation Summary Report (Alberta Health, 2011)
16. 2010-2015 Health Plan Improving Health for All Albertans (Alberta Health Services, 2010)
17. Alberta Health Services Strategic Direction 2009 - 2012 (Alberta Health Services, 2009)
18. Primary Care Networks Governance Framework (Alberta Health, 2017)

ON:

1. Ontario's Action Plan For Health Care 2012

2. Association of Ontario Health Centres: Strategic plan 2012-2015
3. Health Quality Ontario: A Primary Care Performance Measurement Framework for Ontario 2014
4. Patients First: Action for Health Care 2015
5. Patients first: A proposal to strengthen patient-centred health care in Ontario 2015
6. Health Quality Ontario: Quality in Primary Care 2015
7. Health Quality Ontario: Primary Care Performance Measurement: Priority Measures for System and Practice Levels 2015
8. Association of Ontario Health Centres: Championing Transformative Change, Strategic Plan 2015-2020
9. Patients First: Reporting back on the proposal to strengthen patient-centred health care in Ontario 2016
10. Ontario Primary Care Council: Framework for Primary Care in Ontario 2016
11. Health Quality Ontario: A three-year strategic plan 2016-2019
12. Association of family health teams of Ontario: Strategic plan 2017-2020
13. Guide to the advanced health links model
14. Ontario College of Family Physicians: Strategic plan 2018-2021
15. Ontario Health Teams: Guidance for health care providers and organizations
16. Central East Local Health Integration Network Integrated Health Service Plan (2016-2019)
17. Central East LHIN IHSP (2013-2016)
18. Central East LHIN IHSP (2010-2013)
19. Central LHIN IHSP (2016-2019)
20. Central LHIN IHSP (2013-2016)
21. Central LHIN IHSP (2010-2013)
22. Central West LHIN (2016-2019)
23. Central West LHIN (2013-2016)
24. Champlain LHIN (2016-2019)
25. Champlain LHIN (2013-2016)
26. Champlain LHIN (2010-2013)
27. Erie St Clair LHIN IHSP (2016-2019)
28. Erie St Clair LHIN IHSP (2013-2016)
29. Erie St Clair LHIN IHSP (2010-2013)
30. Hamilton Niagara Haldimand Brant LHIN IHSP (2016-2019)
31. Hamilton Niagara Haldimand Brant LHIN IHSP (2013-2016)
32. Hamilton Niagara Haldimand Brant LHIN IHSP (2010-2013)
33. Mississauga Halton LHIN IHSP (2016-2019)
34. Mississauga Halton LHIN IHSP (2013-2016)
35. Mississauga Halton LHIN IHSP (2010-2013)
36. North East LHIN IHSP (2016-2019)
37. North East LHIN IHSP (2013-2016)
38. North East LHIN IHSP (2010-2013)
39. North Simcoe Muskoka LHIN IHSP (2016-2019)
40. North Simcoe Muskoka LHIN IHSP (2013-2016)
41. North West LHIN IHSP (2016-2019)
42. North West LHIN IHSP (2013-2016)

43. North West LHIN IHSP (2010-2013)
44. South East LHIN IHSP (2016-2019)
45. South East LHIN IHSP (2013-2016)
46. South East LHIN IHSP (2010-2013)
47. South West LHIN IHSP (2016-2019)
48. South West LHIN IHSP (2013-2016)
49. South West LHIN IHSP (2010-2013)
50. Toronto Central LHIN IHSP (2016-2019)
51. Toronto Central LHIN IHSP (2013-2016)
52. Toronto Central LHIN IHSP (2010-2013)
53. Waterloo Wellington LHIN IHSP (2016-2019)
54. Waterloo Wellington LHIN IHSP (2013-2016)
55. Wellington Waterloo LHIN IHSP (2010-2013)

QC:

1. Ministère de la santé et des services sociaux - Projet clinique. Cadre de référence pour les réseaux locaux de services de santé et de services sociaux (2004)
   (Ministry of health and social services (MHSS) - Clinical project. Framework for local networks of health services and social services (LHN))
2. Ministère de la santé et des services sociaux - (Loi 10 de 2015) LOI MODIFIANT L'ORGANISATION ET LA GOUVERNANCE DU RÉSEAU DE LA SANTÉ ET DES SERVICES SOCIAUX NOTAMMENT PAR L'ABOLITION DES AGENCES RÉGIONALES - Mise à jour le 12 Juin 2018
   (Ministry of health and social services - (Bill 10 of 2015) An Act to modify the organization and governance of the health and social services network, in particular by abolishing the regional agencies - updated on the 12 June 2018)
3. Ministère de la santé et des services sociaux - Programme de financement et de soutient professionnel pour les groupes de médecine de famille (June 2017)
   (Ministry of Health and Social Services - Funding and Support Program for Family Medicine Groups (June 2017))
4. CISSS Montérégie: Le cadre de référence sur la pratique santé mentale adulte (SMA) en première ligne orientée vers le rétablissement (2015)
   (Montérégie IHSSCs: The Adult Mental Health (ADM) Reference Framework for primary health care oriented on rehabilitation (2015))
5. Cadre de référence de l'approche de partenariat entre les usagers, leurs proches et les acteurs en santé et en services sociaux (2018)
   (Terms of Reference for the Partnership Approach between users, their relatives and actors in health and social services - 2018)
6. MSSS Cadre de référence pour la prévention et la gestion des maladies chroniques physiques en première ligne 2012
   (MSSS Reference Framework for the Prevention and Management of Physical Chronic Disease in the primary health care 2012)
7. MSSS Cadre de gestion des groupes de médecine de famille universitaires (GMF-U)
   (MSSS Management Framework for University Family Medicine Groups (GMF-U))
8. Agence de Santé et des services socauix (ASSS) Mauicie et du Cantre du Québec (MCQ) - Cadre de référence Programme de soutien à la famille pour les personnes ayant une

déficience (2014)
(Mauricie and Quebec Central Agency for Health and Social Services (AHSS) - Terms of Reference Family Support Program for Persons with Disabilities (2014))

9. MSSS Cadre de référence Les ressources intermédiaires et les ressources de type familial. Direction general des services sociaux 2016
   (MSSS Terms of reference Intermediate resources and family-type resources . Directorate general of social services 2016)
10. Vers une meilleure intégration des services pour les jeunes en difficulté et leur famille. Orientations ministérielles relatives au programme-services destiné aux jeunes en difficulté 2017-2022
    (Towards better integration of services for troubled youth and their families. Ministerial Guidelines for the 2017-2022 Youth Services Program)
11. PLAN STRATÉGIQUE du ministère de la Santé et des Services sociaux du Québec 2015-2020. Mise à jour 2017
    (STRATEGIC PLAN of the Quebec Ministry of Health and Social Services 2015-2020. Updated 2017)
12. Pratique clinique de l’infirmière praticienne spécialisée en soins de première ligne : Lignes directrices
    (Clinical Practice of Primary Care Nurse Practitioners: Guidelines)
13. Relever le défi de la maladie d’Alzheimer et des maladies apparentées : Une vision centrée sur la personne, l’humanisme et l’excellence
    (Meeting the Challenge of Alzheimer's Disease and Related Diseases: A Person-Centered Vision, Humanism and Excellence)
14. Un Québec pour tous les âges : Le Plan d’action 2018-2023 ….
    (Quebec for all ages: The 2018-2023 Action Plan)
15. Accès Priorisé Aux Services Spécialisés: Cadre De Référence Régional- Édition mars 2015 (Priority access to specialised services: Regional frame of reference – March 2015 Edition)
