# Supplementary material for: Facilitating Integration Through Team-Based Primary Healthcare: A Cross-Case Policy Analysis of Four Canadian Provinces
Source: Int J Integr Care. 2021 Nov 8;21(4):12. doi: 10.5334/ijic.5680 (PMC8588891; doi:10.5334/ijic.5680)
Supplement: Appendix C. — Data Extraction Table. [file ijic-21-4-5680-s3.pdf]

Appendix C. Data Extraction Table

Source	Scope of policy	Policy Triangle				Principles of Integration									
		Context	Content	Process	Actors	1. Comprehensive services across the care continuum	2. Patient focus	3. Geographic coverage and rostering	4. Standardized care delivery through interprofessional teams	5. Performance management	6. Information systems	7. Organizational culture and leadership	8. Physician integration	9. Governance structure	10. Financial management
